# Evaluating language model embeddings for Parkinson’s disease cohort harmonization using a novel manually curated variable mapping schema

**DOI:** 10.1038/s41598-025-06447-2

**Published:** 2025-06-20

**Authors:** Yasamin Salimi, Tim Adams, Mehmet Can Ay, Helena Balabin, Marc Jacobs, Martin Hofmann-Apitius

**Affiliations:** 1 Department of Bioinformatics, Fraunhofer Institute for Algorithms and Scientific Computing (SCAI), Schloss Birlinghoven 1, 53757 Sankt Augustin, Germany; 2 Laboratory for Cognitive Neurology, Department of Neurosciences, KU Leuven, Leuven, 3000 Belgium; 3 Language Intelligence and Information Retrieval Lab, Department of Computer Science, KU Leuven, Leuven, 3000 Belgium; 4 Bonn-Aachen International Center for IT , Rheinische Friedrich-Wilhelms-Universität Bonn, 53115 Bonn, Germany

**Keywords:** Alzheimer’s disease, Automatic data harmonization, Parkinson’s disease, Large language models, Data stewardship, Data integration, Alzheimer's disease, Parkinson's disease, Functional clustering, Machine learning, Computational models

## Abstract

Data Harmonization is an important yet time-consuming process. With the recent popularity of applications using Language Models (LMs) due to their high capabilities in text understanding, we investigated whether LMs could facilitate data harmonization for clinical use cases. To evaluate this, we created PASSIONATE, a novel Parkinson’s disease (PD) variable mapping schema as a ground truth source for pairwise cohort harmonization using LLMs. Additionally, we extended our investigation using an existing Alzheimer’s disease (AD) CDM. We computed text embeddings based on two language models to perform automated cohort harmonization for both AD and PD. We additionally compared the results to a baseline method using fuzzy string matching to determine the degree to which the semantic capabilities of language models can be utilized for automated cohort harmonization. We found that mappings based on text embeddings performed significantly better than those generated by fuzzy string matching, reaching an average accuracy of over 80% for almost all tested PD cohorts. When extended to a further neighborhood of possible matches, the accuracy could be improved to up to 96%. Our results suggest that language models can be used for automated harmonization with a high accuracy that can potentially be improved in the future by applying domain-trained models.

**Supplementary Information:**

The online version contains supplementary material available at 10.1038/s41598-025-06447-2.

## Introduction

With the rapid growth of collected cohort studies in the field of neurodegenerative diseases, particularly Alzheimer’s and Parkinson’s disease, achieving data interoperability across cohorts has become a significant challenge. Cohort studies typically employ their own naming conventions for collecting measurements and do not adhere to a standard naming system^1^. This leads to a very time-consuming data preprocessing step that would be essential for data-driven analysis across cohorts. Moreover, the set of measurements collected in each cohort study often depends on the specific research questions for which the study was designed^1,2^. As a result, not all cohort studies focus on the same biomarkers or measurements. Given these challenges, it becomes essential to create a Common Data Model (CDM) that considers the variable naming convention of cohorts and demonstrates the availability of the measurements on a granular variable level. Such concerns were previously addressed in Alzheimer’s disease (AD) research. Previous harmonization efforts have resulted in cross-cohort mappings for AD datasets. Establishing variable mappings across cohorts not only allows for assessing the availability of measurements but also lays the groundwork for cohort data interoperability. As a result, such mappings were used to query cohort studies based on measurement availability via the ADataViewer (https://adata.scai.fraunhofer.de/*)* platform (StudyPicker function)^1^. The StudyPicker has been used to identify potential cohorts for cross-cohort analyses based on scientific questions and the corresponding required biomarkers^3^. To our knowledge, such an extensive mapping schema has not been developed for Parkinson’s disease (PD) data, except for a cross-site data model focusing on neuropathological reports within the NeuroBioBank (NBB)^4^. Given the importance of data interoperability for disease investigation and the diversity of unharmonized PD cohorts, a standardized variable mapping schema is crucial for data integration and comparative analysis. Considering patient privacy and data sharing restrictions, the adoption of such a schema into a CDM could significantly accelerate research areas, particularly in the context of federated learning systems^5^.

The person responsible for the harmonization, maintenance, and implementation of FAIR^6^ principles of data in an organization is commonly the position of the chief data officer or data steward^7^. While such curation efforts are important and necessary, they consume a considerable amount of time and resources. Automated^8,9^ or assisted^10,11^ data harmonization therefore has been an ongoing topic of research to facilitate the data harmonization process and improve the efficiency, accuracy, and overall effectiveness of integrating diverse datasets from disparate sources. This ultimately enables seamless collaboration and informed decision-making in various fields, such as healthcare and finance, and fosters scientific cross-cohort research.

Pre-Trained Language Models (PLMs) and in particular Generative Pre-Trained Transformer (GPT) models have shown promising results in various Natural Language Processing (NLP) tasks, including text generation, translation^12^, summarization^13,14^, question-answering^15,16^, and sentiment analysis^17^. They have demonstrated notable capabilities in generating coherent, contextually relevant responses and capturing intricate language patterns, especially for language models at scale (i.e., > 100B parameters), as in the case of large language models (LLMs). While LLMs, and specifically implementations like ChatGPT, have already been proposed to support tasks such as data management^18,19^, documentation^19–21^, or decision finding^22,23^ in a clinical setting, no efforts have yet been made to utilize their capabilities in data harmonization or data stewardship use case. To fill this gap and evaluate the potential of such models to improve data harmonization, we employed a novel PD variable mapping schema and used it as a base to benchmark the performance of an automated mapping approach that utilizes text embeddings. We discuss the potential of automated harmonization approaches using language models and compare them with a commonly used string matching based baseline approach to illustrate the potential merit of utilizing LLMs for automated data harmonization.

## Materials and methods

### Evaluated PD datasets

To establish a consistent structure across diverse cohort studies, developing a CDM could lay the groundwork for data interoperability. Given that no efforts have been made to create a mapping schema for PD to date, we took the first step in developing a CDM. We collected data dictionaries from six distinct PD cohort studies to create a PD variable mapping schema. We manually compared the variables collected in each cohort (Table 1) and established a mapping for variables common across a minimum of two cohorts through manual curation. We continued this procedure until all common variables were mapped across all cohorts. We created a reference term for each variable based on how variables were commonly described in the literature.

**Table 1:** Underlying sources that were used to generate the PD CDM.

Variable source		
Cohort	BIOFIND^38^	The Fox Investigation for New Discovery of Biomarkers in Parkinson’s Disease
	LCC^39^	LRRK2 Cohort Consortium
	LuxPARK^40^	The Luxembourg Parkinson’s Study
	OPDC^41^	Oxford Parkinson’s Disease Centre Discovery Cohort
	PPMI^42^	Parkinson’s Progression Markers Initiative
	PRoBaND^43^	Tracking Parkinson’s
Other	OHDSI^26^	The Observational Health Data Sciences and Informatics
	Ontology^44^	-

A common tool to help the creation of mappings between variables is the OHDSI mapping tool (USAGI)^24^. Another widely used tool to explore ontology terms and their relationships is the Ontology Lookup Service (OLS)^25^. Both tools use the same Apache Lucene-based concept similarity mapping and support widely accepted ontologies like SNOMED CT. Although mapping higher-level variables was relatively straightforward, the number of available target concepts decreased for more granular and specific variables. For instance, while it was feasible to map the total score of a clinical test, often there was no appropriate target for individual question scores within the test. We also chose to use OLS to establish mappings amongst the cohorts’ variables for its broader interoperability beyond OMOP. Additionally, OLS is in active development and provides regular updates.

We semantically harmonized the mappings of all cohorts against the Observational Health Data Sciences and Informatics (OHDSI) Standardized Vocabularies^26^ and mapped them to ontologies that provided respective semantic context, where applicable. The list of included ontologies is reported in Supplementary Table S1 online. We included descriptions for each reference term by extracting from ontology descriptions or obtained through an extensive literature search using standard search engines, including Google Scholar. Lastly, we populated our mapping schema by including additional variables from each cohort, guided by literature findings and focusing on variable importance. We deliberately excluded certain site-specific variables (e.g., assessment date, site number) and instead focused on including biomarkers that were both collected and of interest for data-driven disease investigation. The rationale behind our decision for the inclusion and exclusion of cohort variables was based on those containing measurements rather than indicating whether a test was performed (i.e., yes or no).

We mapped variables that were semantically equivalent across cohorts. Due to the common practice in cohort studies of reporting variables in different ways (e.g., the biological sex of participants: Female, F, 0), we considered these variables as a match. This was done despite the variations in how the variable was recorded, as long as comparability could be achieved with minimal preprocessing steps.

### Investigated AD datasets

To extend our evaluation to a use case beyond PD, we utilized previously established AD variable mappings (i.e., AD-Mapper^8^) for this analysis. We selected 13 cohorts that were included in the AD-Mapper based on the availability and usability of their data dictionaries (Supplementary Table S2, Fig. S1 online). Using the data dictionary of each cohort, we performed the semantic harmonization of the cohort’s variables to the reference terms of the AD-Mapper by applying the harmonization workflow and methods which we elaborate on in the following sections.

### Harmonization workflow

To evaluate the potential merit of an automated harmonization approach based on LLM embeddings, we evaluated two different harmonization strategies using embedding similarity as well as a simple string-matching comparison as a baseline approach. We assessed all strategies by harmonizing each cohort from our novel PD variable mapping schema as well as pre-existing mappings in the context of AD. As variable names alone were often not self-explainable and did not contain enough information to sufficiently describe the semantics of measurements, we instead utilized the variable descriptions that were provided in the data dictionaries of each cohort. For the LCC cohort, we parsed the dictionary tables from the PDF and converted them into CSV format. To prepare a ground truth based on the manually curated variable mappings of both AD and PD for later evaluation of computed mappings, we matched each description from each dictionary to the corresponding variable name in their respective variable mapping source. We were only able to evaluate variables of each cohort where the descriptions were available in the data dictionary since they were needed for both string-matching and the computation of a description embedding. Descriptions in the evaluated data dictionaries were highly diverse in terms of their length and general descriptiveness. We describe this in Supplementary Table S3 online. We manually evaluated the descriptiveness using expert feedback and also reported statistical measures based on word counts to quantify the extent and usability of the different data dictionaries used in this study.

### Mapping evaluation methods

**Fig. 1:**
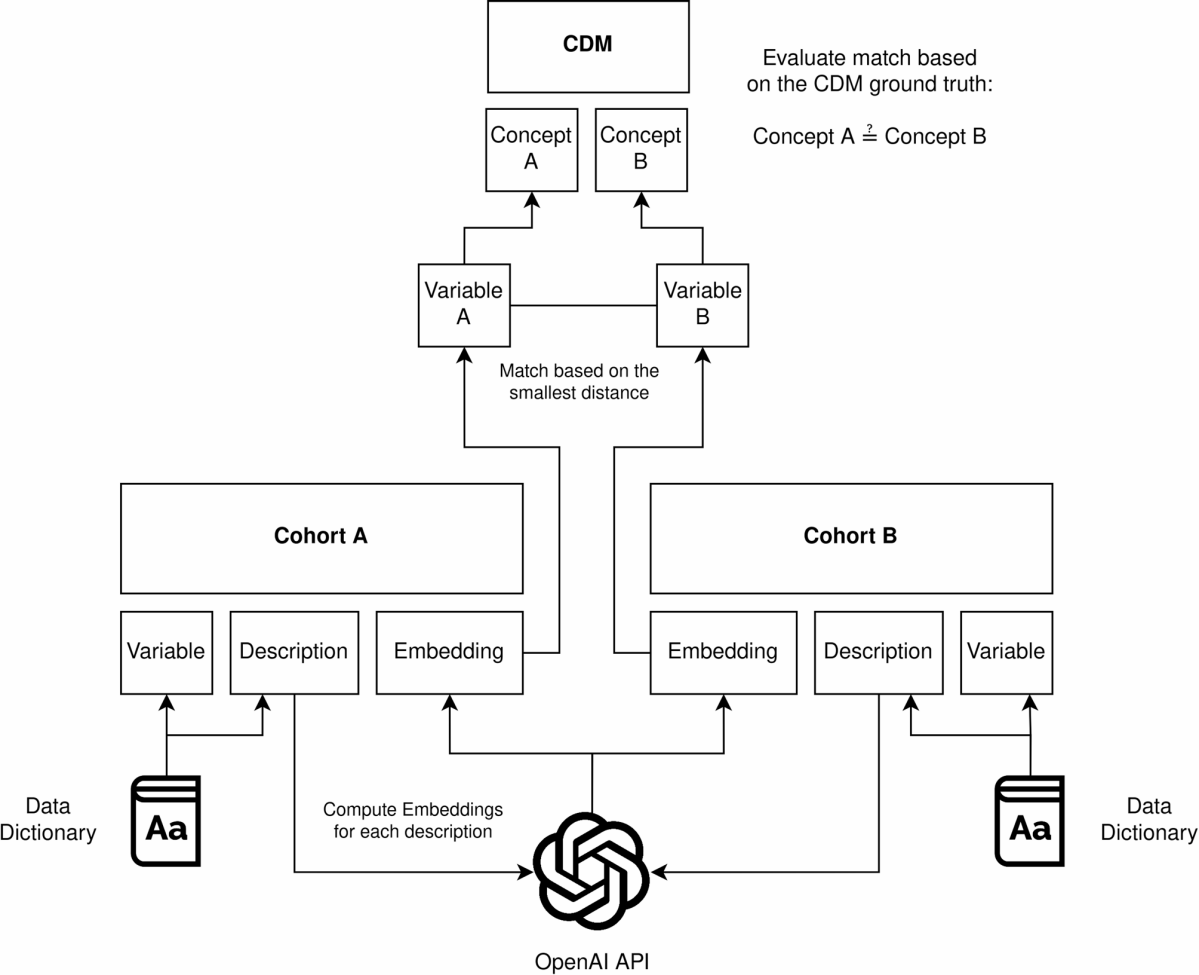
Exemplary workflow for an automatic pairwise harmonization and match evaluation of two different cohorts. Variable descriptions are extracted from the corresponding data dictionaries and embedding vectors are computed via OpenAI API. The vectors are matched based on their smallest distance and the resulting matches are evaluated based on the underlying CDM variable mappings.

As for the first strategy, we evaluated the accuracy of harmonization matches of each pair of cohorts, both within PD and AD, based on the cosine similarity of each available variable description. An exemplary workflow for pairwise harmonization is shown in Fig. 1.

In the first step, variable names and descriptions were extracted from each data dictionary. We then computed sentence text embeddings based on average pooling over token-level embeddings for each description on each cohort variable using two different models. First, we retrieved text embeddings from the OpenAI embeddings API for their most recent (text-embedding-3-large) embedding model^27^. Second, to compare the performance of LLM embeddings on a smaller, local PLM, we also retrieved text embeddings using MPNet^28^. The MPNet embeddings were calculated using the all-mpnet-base-v2 model hosted at Hugging Face. The model was trained on over a billion sentence pairs, resulting in a parameter space comprising more than 110 million parameters^28^. We selected MPNet over other architectures like Bidirectional Encoder Representations from Transformers (BERT) due to its demonstrated improvements in capturing contextual nuances and overall performance^28^. While there are more recent encoder-only models that also surpass BERT, MPNet remains a strong choice for achieving high-quality embeddings in our specific application. Using the computed embeddings, each variable from the source cohort A can be mapped to a single variable in the target cohort B based on the contextual similarity of their variable descriptions. As a measure of similarity, we calculated a cosine similarity matrix containing the similarities of each variable description from cohort A to each possible description match in cohort B. We then matched each variable in cohort A to the corresponding variable with the highest possible similarity in cohort B. Based on the concept labels in the variable mapping source that are provided for all variables for both cohorts, we could then check for equality of the concept labels and assess whether the calculated match was correct or not.

To have a baseline comparison to a more conventional approach, we also computed the closest description matches using fuzzy string matching^29^. The implementation used the calculated *Levenshtein distance*^30^ to rank the description strings based on their single-letter edit distance.

While in the best case, the correct match should also be the closest in terms of description distance, it may be useful to also consider the n closest matches instead. This especially applies if we consider a semi-automated harmonization approach, where a data steward has the option to choose a match from a list of potential candidates. To evaluate this matching strategy, we computed embedding similarities and Levenshtein distances as in the closest match scenario and ranked the 20 best matches by their lowest computed distances.

### Embedding visualizations

To further evaluate which variable descriptions get matched and clustered based on their embedding proximities, we used t-distributed stochastic neighbor embedding (t-SNE)^31^ to compute a two-dimensional representation of each available description embedding for all AD and PD cohorts. We created two plots, one visualizing all available data points color-coded to their affiliation to AD and PD, and the other visualizing their affiliation to their respective cohort through a color-coded label.

## Results

We established a PD variable mapping schema, containing a variable naming system for six cohorts, harmonized against the OHDSI vocabulary and ontology terms, resulting in 739 unique reference terms. Together with our previously established AD CDM^1^, we evaluated pairwise mappings of a total of 19 different cohorts in the context of neurodegenerative diseases.

We employed our variable mapping schema for PD and the existing mappings for AD to benchmark the performance of text embedding-based automatic harmonization, as well as a simple string matching based approach. We calculated embeddings using OpenAIs most recent embedding model API as well as MPNet in a local setting to compute similarities between cohort descriptions for an automated harmonization workflow. A detailed description of our workflow is shown in Fig. 1 in the method section. Our results suggest that automatic harmonization of cohort data can be achieved for both PD and AD cohort studies.

### PASSIONATE

**Fig. 2:**
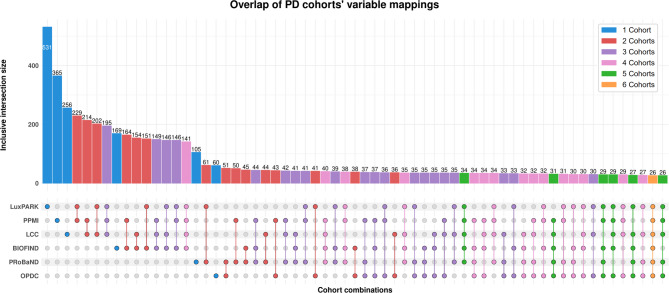
The total number of variable mappings from each cohort, as well as the variable overlap within sets of cohorts. Note: we deliberately excluded any empty sets.

We created PASSIONATE^32^, a PD variable mapping schema comprising cross-mappings of six distinct cohorts. We enriched the variable mappings of PASSIONATE with the OHDSI vocabulary and ontology terms wherever the terms were available. As a result, PASSIONATE comprised 739 unique terms harmonized across eight different sources. The number of included variables from each source (Supplementary Table S4 online) and the number of overlapping variable mappings between combinations of cohorts are reported in Fig. 2. Within PASSIONATE, the total number of variables stemming from each source varied across cohorts. For instance, the total number of variables that were included from the LuxPARK cohort was 531, while only 60 variables of the OPDC cohort could be encompassed (Fig. 2). This was a result of certain cohorts collecting a higher number of variables in comparison to others as well as available descriptions for their variables.

We observed that certain cohorts shared a large number of variables, whereas in other cohorts, the opposite was noted. Among the LuxPARK and PPMI cohorts, 229 variables could be semantically harmonized. LCC and OPDC had the smallest variable overlap with a total of 36 variables. The mapping overlap among sets with three or more cohorts varied across different sets. One notable example of having the most common variables was the overlap among LuxPARK, PPMI, and LCC with a total of 195 variables. Replacing one of those cohorts with another decreased the number of common variables noticeably. For instance, LuxPARK, LCC, and OPDC had 30 common variables that were included in PASSIONATE, and similarly, LuxPARK, LCC, and PRoBaND had 35 variables. We made PASSIONATE publicly available at Zenodo (10.5281/zenodo.10218362).

### Cohort harmonization

By performing semantic harmonization for each pair of cohorts, in addition to harmonizing each cohort against the reference terms of PASSIONATE, we calculated the accuracy of each mapping (Table 2). Our results revealed that in 25 out of 30 cases of pairwise cohort harmonization, our proposed approach based on OpenAI embeddings achieved an accuracy above 80%. Cohort-to-cohort harmonization using MPNet showed an accuracy of above 80% in 18 cases. In contrast, string matching only achieved an accuracy above 80% in six cases. Similarly, when harmonizing each cohort against the reference terms of PASSIONATE (i.e., using their descriptions), using both OpenAI embeddings and MPNet approaches obtained a significantly higher accuracy than string matching for all cohorts. LuxPARK demonstrated the highest string matching accuracy in harmonizing a cohort against the PASSIONATE reference terms, achieving 24%. Utilizing MPNet embeddings increased the accuracy to 66% while employing the OpenAI embeddings further boosted it to 67%.

**Table 2:** Calculated accuracy of the highest similarity match using OpenAI embeddings, MPNet approach, and the closest match using fuzzy string matching. The total number of variables that could have been included in this analysis is reported in parentheses. Note: these numbers are based on the variables’ description availability and existence of the variable within PASSIONATE.

Mapping from / mapping to	Method	OPDC (*n* = 36)	PRoBaND (*n* = 46)	BIOFIND (*n* = 124)	LCC (*n* = 154)	LuxPARK (*n* = 189)	PPMI (*n* = 210)	PASSIONATE (*n* = 320)
OPDC	Fuzzy	-	48%	9%	50%	29%	20%	0%
	MPNet	-	100%	77%	65%	71%	80%	58%
	OpenAI embeddings	-	100%	73%	95%	88%	80%	56%
PRoBaND	Fuzzy	74%	-	15%	36%	43%	24%	13%
	MPNet	100%	-	77%	76%	70%	81%	63%
	OpenAI embeddings	100%	-	69%	88%	90%	76%	61%
BIOFIND	Fuzzy	41%	30%	-	93%	68%	91%	11%
	MPNet	94%	87%	-	96%	81%	96%	59%
	OpenAI embeddings	94%	91%	-	95%	87%	97%	65%
LCC	Fuzzy	53%	42%	88%	-	69%	88%	19%
	MPNet	84%	88%	91%	-	84%	93%	59%
	OpenAI embeddings	89%	92%	93%	-	90%	92%	64%
LuxPARK	Fuzzy	52%	33%	63%	68%	-	60%	24%
	MPNet	59%	67%	67%	71%	-	69%	66%
	OpenAI embeddings	56%	69%	90%	89%	-	84%	67%
PPMI	Fuzzy	33%	38%	89%	88%	65%	-	22%
	MPNet	87%	90%	92%	96%	78%	-	62%
	OpenAI embeddings	87%	90%	96%	97%	84%	-	71%

**Fig. 3:**
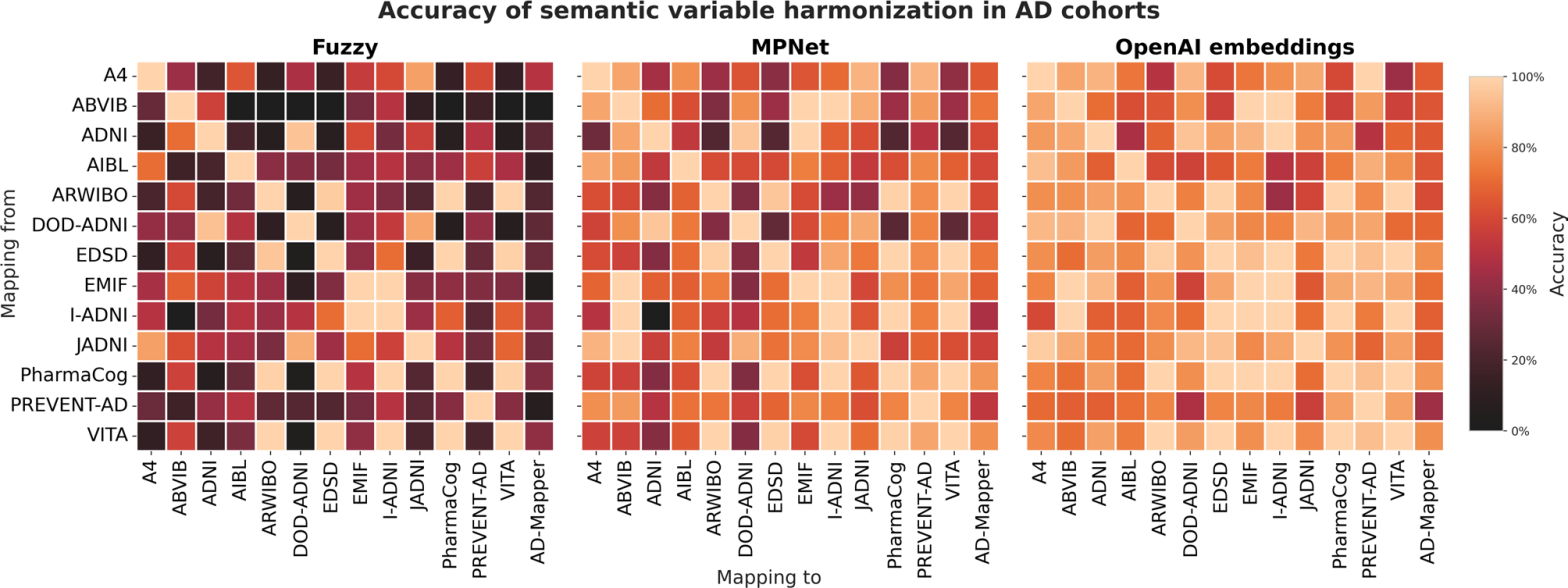
The accuracy of semantic harmonization of AD cohorts’ variables using string matching and OpenAI and MPNet embeddings. The individual accuracies are reported in Supplementary Tables S4-S6 online.

We performed the same investigation using the previously established variable mappings for AD cohorts (i.e., AD-Mapper). We conducted semantic harmonization of 13 distinct AD cohort studies once utilizing OpenAI and MPNet embeddings and fuzzy string matching as a baseline approach. The accuracies of all strategies are presented in Fig. 3 and the individual values are shown in Supplementary Tables S5-S7 online. Due to the volume of results, individual comparison was impractical. Therefore, we implemented a ranking system to assess model accuracy, indicating superior performance within a comprehensive table (Supplementary Table S8 online). Similarly to the previous experiment, OpenAI and MPNet embeddings resulted in much higher accuracy than the string matching approach (Fig. 3). In 102 cases, GPT demonstrated superior accuracy, followed by MPNet in 18 cases (Supplementary Fig. S2 online). Additionally, in 37 cases, both OpenAI and MPNet embeddings exhibited equal accuracies, all surpassing the performance of string matching. The remaining 8 cases did not show a clear preference (i.e., equal accuracy), while in 4 cases, string matching and OpenAI outperformed the MPNet embeddings. Lastly, using string matching for variable harmonization showed 0% accuracy in 8 cases (e.g., harmonizing Aging Brain: Vasculature, Ischemia, and Behavior (ABVIB)^33^ to Alzheimer’s Disease Repository Without Borders (ARWIBO)^34^).

**Fig. 4:**
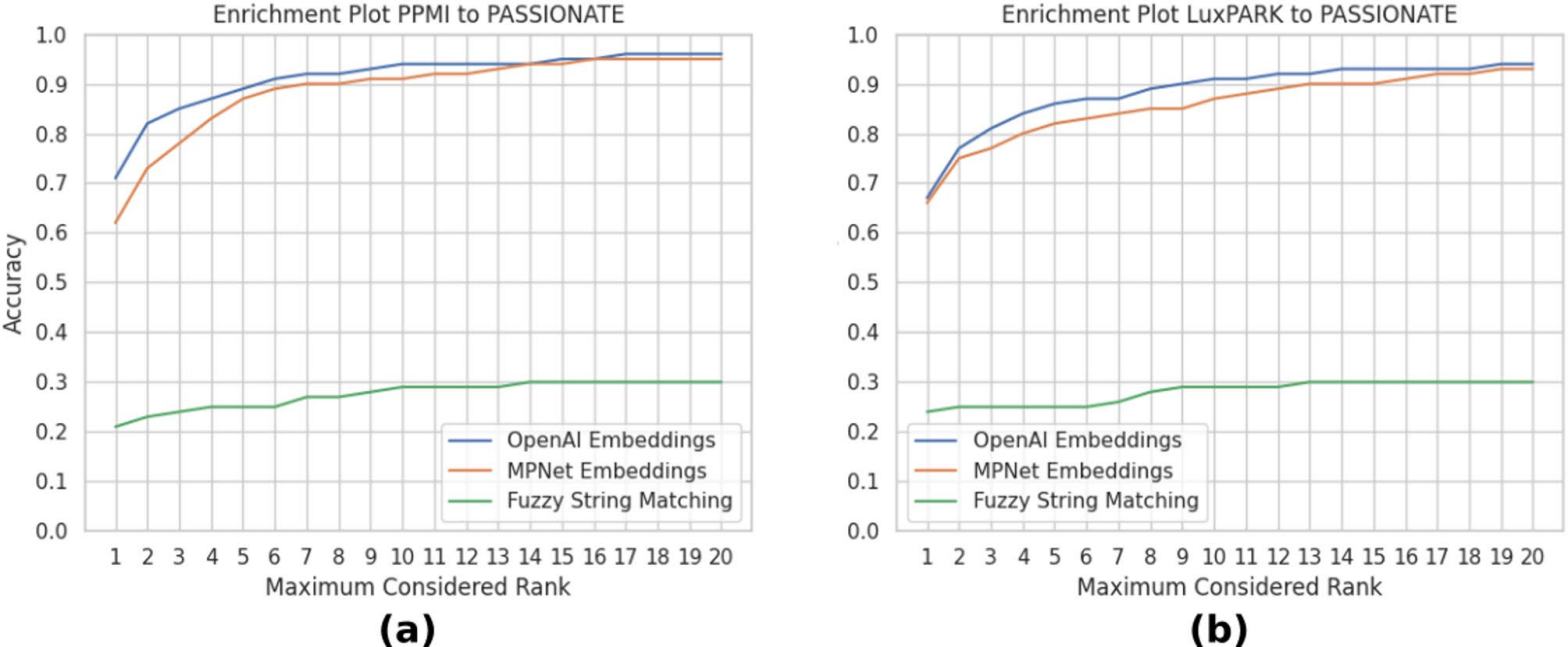
Accuracy for correctly matching the highest n-ranked variables for the OpenAI, MPNet, and fuzzy approaches in the pairwise mappings of (**a**) PPMI and PASSIONATE and (**b**) LuxPARK and PASSIONATE.

To evaluate to which degree the consideration of lower-ranked matches can potentially improve the accuracy of a (semi)-automated mapping, we inspected the k-nearest neighbors in terms of their highest cosine similarity up to a maximum rank of 20. We chose to evaluate the mapping from both PPMI and LuxPARK cohorts to PASSIONATE, as both contained the most variables out of all six evaluated PD cohorts and the automatic mapping (cf. Table 2) had the lowest accuracy when mapping to PASSIONATE. The results from this enrichment approach are shown in Fig. 4.

For the mapping from PPMI to PASSIONATE (Fig. 4a), we noticed that by considering each second closest neighbor, the overall accuracy improved for the embedding-based matching by over 10% from 71% to 82%. When considering additional matches, the accuracy could be further improved up to 96% for a considered rank of 16, while it did not improve any further in our evaluation of a rank up to 20. While we observed a similar increase in accuracy for the fuzzy string matching approach, the increase in accuracy was lower and capped earlier to a maximum of 30% at a rank of 14. Similarly, the embeddings based on the MPNet model also outperformed fuzzy string matching by a large margin, while mappings based on OpenAI embeddings still performed significantly better for most ranks, particularly for lower-ranking neighborhoods. We found a similar result for the mapping of LuxPARK to PASSIONATE (Fig. 4b), where for the embedding-based mapping, considering the second closest match improved the accuracy from 67% to 77%, capping out at 94% at rank 19. The MPNet embeddings again performed similarly well with a starting accuracy of 66% at rank 1, capping out at 93% at rank 19. The fuzzy string matching results also improved similarly to the mapping from PPMI, capping out at an accuracy of 30% at rank 13.

### Clustering of cohort variable descriptions

**Fig. 5:**
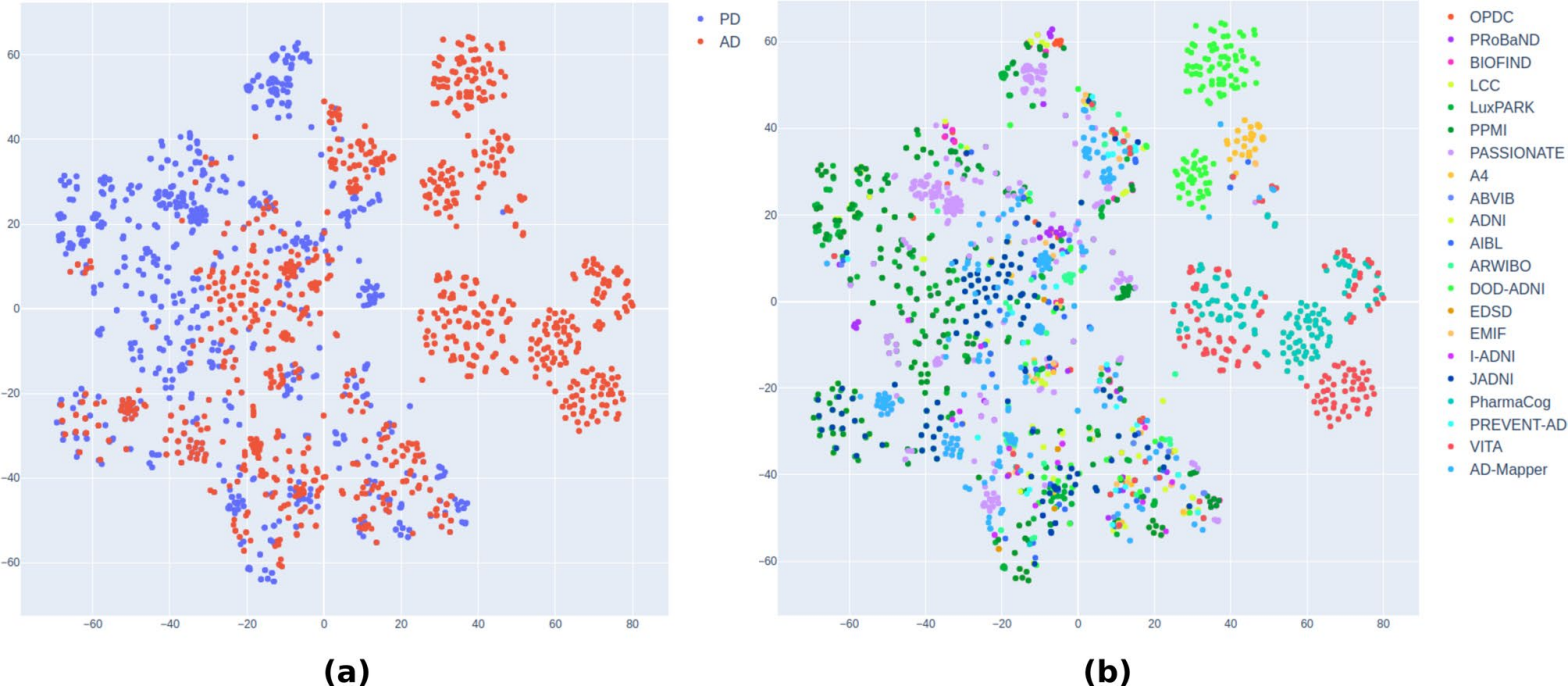
Two-dimensional t-SNE distribution plot of all computed AD and PD variable description embeddings using OpenAI. The first plot (**a**) shows the distribution between AD and PD variable descriptions, and the second plot (**b**) the distribution of each cohort. While some cohort variables form homogeneous clusters for their cohort-specific descriptions, other clusters are comprised of variable descriptions from different cohorts with the same underlying semantics. The same analysis has been done using MPNet embeddings and can be seen in Supplementary Figure S3 Online.

We utilized t-SNE to illustrate the embeddings of variable descriptions across all examined cohorts. Figure 5a shows the distribution of the embeddings computed from all available PD cohort variable descriptions (blue) versus the distribution of all available AD cohort descriptions (red). As expected, we observed the formation of clusters within each disease’s variable descriptions, as well as distinct clusters within each disease. However, we noticed a significant overlap in variable descriptions between both disease types, as depicted in the central cluster in Fig. 5a.

In the second visualization, Fig. 5b, we color-coded each embedding according to its cohort origin, regardless of its association with AD or PD. We found again that some variable clusters are cohort-specific (e.g. the two top side clusters belonging to Effects of the Study of Brain Aging in Vietnam War Veterans (DOD-ADNI)^35^), while others are formed by a mixture of different cohorts. We host an interactive version of Fig. 5b (https://tsnepad.scai.fraunhofer.de), enabling users to toggle the visibility of specific cohorts and view the underlying variable description by hovering the mouse over the corresponding data point.

## Discussion

The semantic harmonization of six PD cohort studies through manual curation revealed two major aspects. Heterogeneity was observed regarding the granularity of variables within these cohorts. Different cohorts collected specific sets of variables, which were not always common across the investigated cohorts. This was not surprising, as cohort studies often collected measurements based on the goals of their studies^2^. However, as such cohorts are often the basis for data-driven analysis, it was vital to investigate the variable overlap to determine potential cohorts that could be used as training and validating datasets^36^. For instance, among the variable mappings done within PASSIONATE, LCC, and OPDC, cohorts shared 36 variables. The naming conventions and the way that measurements were reported among investigated datasets differed substantially in most cases, except for PPMI and BIOFIND. This difference resulted from cohort studies often employing their naming conventions for collecting measurements. Although similarities were observed among certain variables (e.g., the variable “gender” in LCC, PRoBaND, and OPDC), that was not the case for the majority of variables. Nevertheless, by generating PASSIONATE, we emphasized the significance of a standardized variable mapping in light of the limitations inherent in cohort studies. Such a mapping schema could prove beneficial, particularly when employed for data-driven analyses and investigations using federated learning.

We conducted semantic harmonization of AD and PD cohorts’ variables by analyzing the similarities in the embeddings of variable descriptions generated using OpenAI and MPNet embeddings in addition to the string matching approach. The results of our experiment indicated that OpenAI-based embeddings yielded the highest accuracy, followed by the MPNet approach, with the string matching method being the least accurate. While OpenAI embeddings considerably outperform MPNet embeddings in several cases, the difference is not proportional to the substantial disparity in model capacity. MPNet already performs the mapping task well for most pairwise PD cohort mappings, with the OpenAI embeddings not providing additional value in several cases. This could be explained by the fact that the MPNet performance of the mapping accuracy is already high, leaving little room for improvement in several cases. Additionally, both methods strongly rely on the quality of variable descriptions, with short or inadequate descriptions potentially not allowing OpenAI embeddings to improve the smaller model’s performance to its full potential. This is particularly evident in the evaluated AD mappings. In pairwise mappings for AD cohorts, although the accuracies based on OpenAI and MPNet were significantly higher than string matching, they achieved an accuracy above 80% in only 85 and 48 out of 156 cases. String matching, on the other hand, achieved an accuracy above 80% in only 22 cases. This could be the result of AD cohorts’ descriptions not being sufficiently descriptive. For instance, in the Italian Alzheimer’s Disease Neuroimaging Initiative (I-ADNI)^37^ cohort, the cohort’s dictionary lacked information on all available variables, and the descriptions were not informative. We also found that for many cohorts, the length of descriptions did not correlate with the actual quality of the descriptions (see Supplementary Table S3 online).

The harmonization of each PD cohort against PASSIONATE exhibited the lowest accuracy for all approaches, except for two cases where MPNet and OpenAI embeddings were used to harmonize LuxPARK to OPDC. This could potentially be the result of dissimilarities between PASSIONATE descriptions and the description of variables in each cohort, given that PASSIONATE descriptions were extracted from ontologies. While harmonizing the AD cohort to the AD-Mapper variables, the accuracies varied and were not consistently the lowest. Nevertheless, utilizing OpenAI embeddings demonstrated a substantially higher accuracy than fuzzy string matching, with MPNet emerging as the second-best option. The accuracies we computed in Table 2 and Supplementary Tables S5-S7 online are non-symmetric for most pairwise mappings. This is due to the fact that the mappings contained upper-level concepts for several of the evaluated cohorts. As a result, one cohort mapping may have higher accuracies due to correct matches to the upper-level concept in the other, while in the reversed matching case such an upper-level concept may not be present. For more details refer to the supplementary material online (Accuracy: A to B vs. B to A).

Based on the results shown in Fig. 4, it is apparent that even though the automated OpenAI-based mapping results perform notably well with an accuracy of over 80% for most of the pairwise PD mappings (Table 2), considering lower-ranked matches can further enhance the harmonization accuracy by a considerable amount. Given that the inclusion of the second match already improves performance by over 10% and the total accuracy can be enhanced to 96% when considering up to 16 closest matches, the merit of this approach becomes very apparent. Data Harmonization is often approached in a semi-automatic process, where the Data Steward will actively orchestrate curation with the support of Data-Stewardship Tools (DST)^10,11^. One way to take advantage of a higher mapping accuracy of several close OpenAI embedding matches would be to provide the Data Steward with a list of optimal suggestions for mapping. It could also be possible to threshold the length of such a list based on the maximum allowed embedding distance. This would ensure that the Data Steward receives the most optimal and relevant number of recommendations without introducing an overhead of too many suggestions for manual curation.

The matching of embeddings can inherently be seen as a clustering task. Variables with a small semantic embedding distance from each other form semantic clusters when they describe a common concept. The projection of variable description embeddings revealed significant overlap between AD and PD cohorts (Fig. 5a), with clusters emerging across various modalities, indicating similarities in variable space. However, distinct clusters formed within each disease type, suggesting semantic dissimilarity, likely due to variations in data collection across cohorts. For instance, MRI measurements were not included in the six PD cohort studies analyzed. Additionally, encoding description embeddings with cohort origin (color-coded) revealed mixed clusters, indicating potential harmonization opportunities. Variables with similar descriptions were closely located, suggesting harmonization potential within reference terms and associated cohorts.

One major limitation of our approach was the requirement for descriptions (e.g., in the form of data dictionaries) to compute fitting mapping candidates. As the information solely present in variable names was very limited, we found it unsuitable as a basis for any informative harmonization approach in most cases. Consequently, variables that did not provide descriptions could not be harmonized, leading to their exclusion from our evaluation. In the AD use case, we were obliged to exclude several cohorts that did not provide descriptive information or offered supplementary data (i.e., a data dictionary) in a format that was irretrievable, such as having ambiguous Unicode encodings due to different language formats. We do, however, expect our OpenAI-based mapping approach to potentially harmonize cohorts with data dictionaries in different languages with a similar performance as demonstrated in this paper. This assumption still needs validation and is part of our current research. While we could potentially harmonize variables based on their descriptions, our harmonization approach is meant to rely on a baseline of pre-curated data. Without a baseline model in the form of a CDM or a curated terminology, even though variables could be mapped against each other, their mapping validity would be hard to assess. Lastly, the PASSIONATE is limited to variables collected in the investigated PD cohorts and, as such, does not represent the entire PD variable landscape.

In this work, we primarily focused on mapping metadata to standardized vocabulary. Another possible extension of the current approach would be to extend our variable mapping schema to allow users to directly map their data into the Observational Medical Outcomes Partnership (OMOP) format. However, implementing automatic mapping to OMOP is complex and requires extensive data validation to ensure consistency across diverse data sources, which we have not yet fully addressed. We are currently working on such an implementation.

## Conclusion

Given that cohort data harmonization is essential and continues to be an area of ongoing research, and considering that cohort studies often serve as the foundation for data-driven analysis, we developed PASSIONATE, a PD variable mapping schema, and have made it publicly available. By leveraging PASSIONATE and previously established AD mappings, we assessed the feasibility of automated variable harmonization across cohorts. Both tested language models outperformed string matching by a large margin, as expected. While both models perform considerably well, especially when considering clusters of potential matches, they are general models not fine-tuned for biomedical data. Fine-tuning these models for biomedical data could potentially further enhance the already promising results. Our work demonstrates the potential of using advanced language models for cohort data harmonization, highlighting the importance of model customization to fully leverage their capabilities. Our mapping schema provides a valuable resource for the PD research community, facilitating more efficient and accurate data harmonization in future studies and cross-cohort analyses.

## Electronic supplementary material

Below is the link to the electronic supplementary material.

Supplementary Material 1

## Data Availability

The datasets generated and/or analyzed during the current study are not publicly available due to patient data privacy concerns but are available from the corresponding author upon reasonable request. The cohort’s data dictionaries analyzed during the current study are available in the following repositories: A4, ABVIB, ADNI, AIBL, DOD-ADNI, PPMI, and BIOFIND (https://ida.loni.usc.edu/login.jsp); LCC (https://www.michaeljfox.org/news/lrrk2-cohort-consortium); LuxPARK (request.ncer-pd@uni.lu); OPDC (https://portal.dementiasplatform.uk/CohortDirectory/Item? fingerPrintID=OPDC%20Discovery); ProBaND (https://www.dpag.ox.ac.uk/opdc/team/proband-tracking-parkinsons); ARWIBO, EDSD, I-ADNI, PharmaCog, and VITA (https://www.neugrid2.eu); EMIF (https://www.emif.eu/emif-ad-2/); JADNI (https://humandbs.dbcls.jp/en/hum0043-v1); and PREVENT-AD (https://registeredpreventad.loris.ca/). The code utilized to perform the analyses in this manuscript is available at https://github.com/SCAI-BIO/datastew as well as https://github.com/SCAI-BIO/tsnepad. The generated Parkinson’s disease common data model is publicly available at Zenodo 10.5281/zenodo.10218362.

## Abbreviations

A4: Anti-amyloid treatment in asymptomatic Alzheimer’s disease
ABVIB: Aging brain: vasculature, ischemia, and behavior
AD: Alzheimer’s disease
ADNI: The Alzheimer’s disease neuroimaging initiative
AIBL: The Australian imaging, biomarker & lifestyle flagship study of ageing
ARWIBO: Alzheimer’s disease repository without borders
BIOFIND: The Fox investigation for new discovery of biomarkers in Parkinson’s disease
CDM: Common data model
DOD-ADNI: The study of brain aging in Vietnam war veterans
DST: Data stewardship tool
EDSD: The European DTI study on dementia
EMIF: European medical information framework
GPT: Generative pre-trained transformer
I-ADNI: The Italian Alzheimer’s disease neuroimaging initiative
JADNI: Japanese Alzheimer’s disease neuroimaging initiative
LCC: LRRK2 cohort consortium
LLM: Large language model
LuxPARK: The Luxembourg Parkinson’s study
MRI: Magnetic resonance imaging
NLP: Natural language processing
OHDSI: Observational health data sciences and informatics
OMOP: Observational medical outcomes partnership
OPDC: Oxford Parkinson’s disease centre discovery cohort
PD: Parkinson’s disease
PharmaCog: Prediction of cognitive properties of new drug candidates for neurodegenerative diseases in early clinical development
PLM: Pre-trained language model
PPMI: Parkinson’s progression markers initiative
PREVENT-AD: Pre-symptomatic evaluation of experimental or novel treatments for Alzheimer’s disease
PRoBaND: Tracking Parkinson’s
t-SNE: t-distributed stochastic neighbor embedding
VITA: Vienna transdanube aging
