# Supplementary material for: Evaluating language model embeddings for Parkinson’s disease cohort harmonization using a novel manually curated variable mapping schema
Source: Sci Rep. 2025 Jun 20;15:20210. doi: 10.1038/s41598-025-06447-2 (PMC12181335; doi:10.1038/s41598-025-06447-2)
Supplement: Supplementary file 1 — Supplementary Material 1 [file 41598_2025_6447_MOESM1_ESM.pdf]

Evaluating Language Model Embeddings for Parkinson's Disease Cohort Harmonization Using a Novel Manually Curated Variable Mapping Schema

Yasamin Salimi[1⁑], Tim Adams[1⁑], Mehmet Can Ay[1], Helena Balabin[2,3], Marc Jacobs[1*], Martin Hofmann-Apitius[1,4]

1. Department of Bioinformatics, Fraunhofer Institute for Algorithms and Scientific Computing (SCAI), Sankt Augustin 53757, Germany
2. Laboratory for Cognitive Neurology, Department of Neurosciences, KU Leuven, Leuven 3000, Belgium
3. Language Intelligence and Information Retrieval Lab, Department of Computer Science, KU Leuven, Leuven 3000, Belgium
4. Bonn-Aachen International Center for IT, Rheinische Friedrich-Wilhelms-Universität Bonn, Bonn 53115, Germany

***Corresponding author:** Dr. Marc Jacobs, Fraunhofer-Institute for Algorithms and Scientific Computing (SCAI), Schloss Birlinghoven 1, 53757 Sankt Augustin, marc.jacobs@scai.fraunhofer.de, +49 - 2241 - 14 - 4013

⁑ The authors contributed equally to this work.

While generating the PD variable mapping schema, we linked variables to ontologies to cross-reference them with controlled vocabularies. Variables were checked against definitions in the Ontology Lookup Service (OLS) (https://www.ebi.ac.uk/ols/index). When multiple matches occurred, we prioritized referential ontologies due to their regular updates and extensive cross-references. The availability of the ontologies was used as a selection criterion for the harmonization step as their descriptions were essential for comparison against the cohorts' variables' descriptions.

Table S1: List of ontologies used in the creation of PASSIONATE.

Ontology acronyms	Ontology Full Names
ADO	Alzheimer's Disease Ontology (ADO)
EFO	Experimental Factor Ontology
FOODON	Food Ontology
GO	Gene Ontology
HP	Human Phenotype Ontology
MONDO	Mondo Disease Ontology
NCIT	NCI Thesaurus OBO Edition
OMIT	Ontology for MIRNA Target
PR	PRotein Ontology
SCDO	Sickle Cell Disease Ontology
SNOMED	SNOMED CT (International Edition)
VO	Vaccine Ontology

Table S2: Investigated AD cohort studies and their total number of variables that were included in AD-Mapper variable mappings.[1] Note: we excluded cohorts that had no description provided in their data dictionaries or contained encodings that were not compatible with our mapping procedure.

Variable origin		Consortium	# Mapped variables
**Cohort**	**A4**[2]	Anti-Amyloid Treatment in Asymptomatic Alzheimer's Disease	73
	**ABVIB**[3]	Aging Brain: Vasculature, Ischemia, and Behavior	12
	**ADNI**[4]	The Alzheimer's Disease Neuroimaging Initiative	340
	**AIBL**[5]	The Australian Imaging, Biomarker & Lifestyle Flagship Study of Ageing	54
	**ARWIBO**[6]	Alzheimer's Disease Repository Without Borders	1104
	**DOD-ADNI**[7]	Effects of TBI & PTSD on Alzheimer's Disease in Vietnam Vets	322
	**EDSD**[8]	The European DTI Study on Dementia	1061
	**EMIF**[9]	European Medical Information Framework	30
	**I-ADNI**[10]	The Italian Alzheimer's Disease Neuroimaging Initiative	1064
	**JADNI**[11]	Japanese Alzheimer's Disease Neuroimaging Initiative	647
	**PharmaCog**[12]	Prediction of Cognitive Properties of New Drug Candidates for Neurodegenerative Diseases in Early Clinical Development	1067
	**PREVENT-AD**[13]	Pre-symptomatic Evaluation of Experimental or Novel Treatments for Alzheimer's Disease	34
	**VITA**[14]	Vienna Transdanube Aging	1054

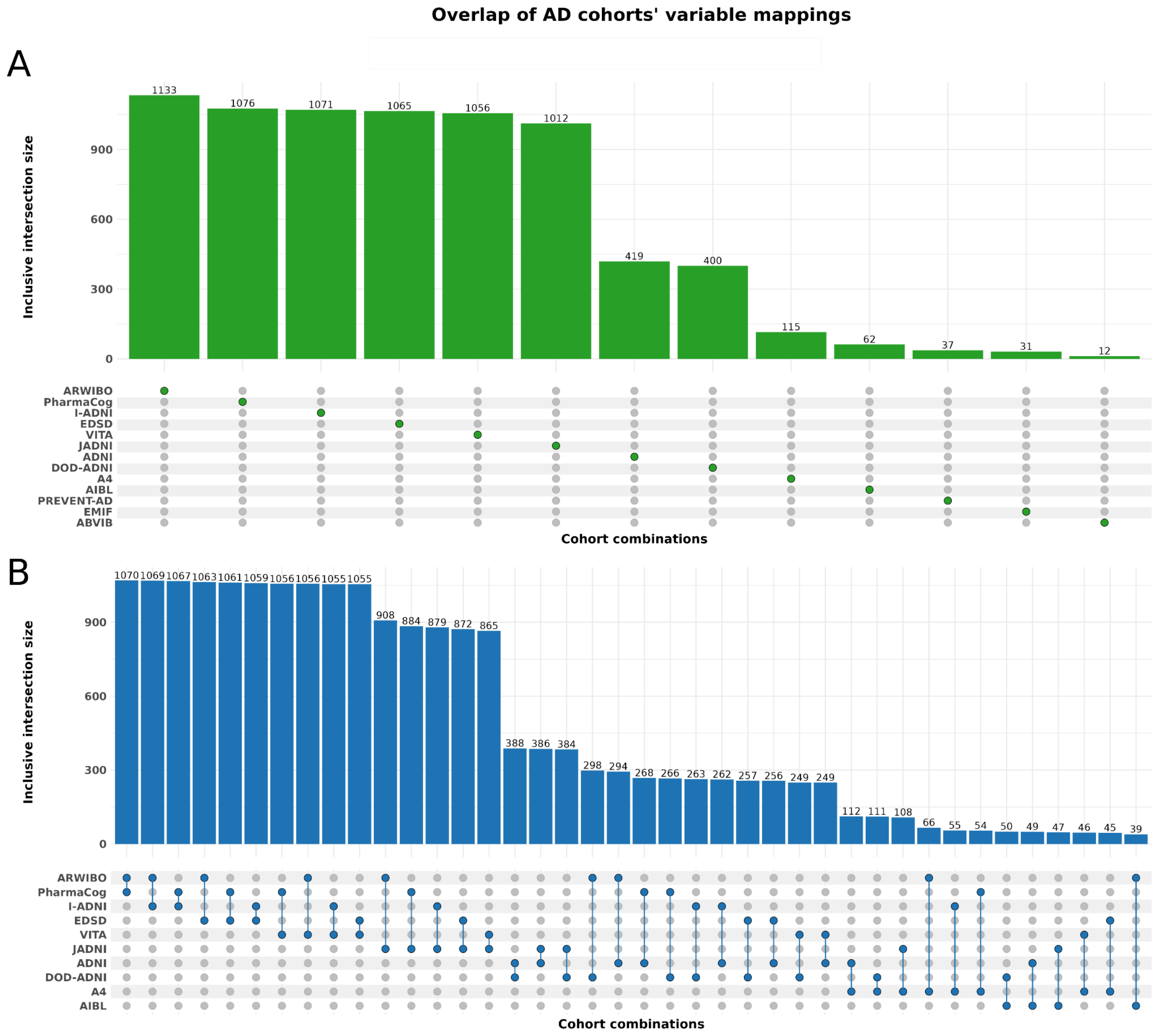

Figure S1: The total number of variable mappings from each cohort (panel A), as well as the variable overlap within two cohorts (panel B). Note: we deliberately excluded the empty sets and certain cohort combinations with variable overlap lower than 30.

Table S3: Average, median, mode, and total number of word counts used to describe variables from the respective dictionaries. We additionally report the quality of description by manual expert curation into categories: *poor* (description is vague, unclear, or missing key details), *adequate* (description is understandable but could be clearer or more detailed), *excellent* (description is clear, precise, and fully informative).

Cohorts		Word Count				Quality Evaluation
		Average	Median	Mode	Total	
**AD**	**A4**	5.5	5	5	539	adequate
	**ABVIB**	11	4	4	121	excellent
	**ADNI**	4.69	5	5	1381	adequate
	**AIBL**	3.51	3	2	193	adequate
	**ARWIBO**	6.59	6	7	1398	poor
	**DOD-ADNI**	5.59	5	5	1381	poor
	**EDSD**	6.06	7	7	951	adequate
	**EMIF**	4.61	4.5	3	129	excellent
	**I-ADNI**	3.19	3	3	51	poor
	**JADNI**	5.37	3	2	655	poor
	**PharmaCog**	6	7	7	919	poor
	**PREVENT-AD**	6.58	6	6	263	excellent
	**VITA**	6.13	7	7	919	poor
**PD**	**BIOFIND**	3.79	4	4	470	poor
	**LCC**	4.67	4.5	5	719	excellent
	**LuxPARK**	5.92	4	2	1118	poor
	**OPDC**	10.08	9.5	6	363	excellent
	**PPMI**	4.1	4	3	860	adequate
	**PRoBaND**	10.91	9.5	4	502	excellent

Table S4: Total number of included variables in PASSIONATE from each source.

Variable Source		# Included variables
Cohort	BIOFIND[15]	146
	LCC[16]	239
	LuxPARK[17]	507
	OPDC[18]	45
	PPMI[19]	336
	PRoBaND[20]	87
Other	OHDSI[21]	201
PASSIONATE	Reference Term	739
	Ontology[22]	266

Table S5: Calculated accuracy of the closest match using fuzzy string matching for all applicable cohorts in the AD-Mapper variable mappings. The n describes the number of variables that have a corresponding mapping in the AD-Mapper variable mappings.

Mapping from / mapping to	Method	A4 (n=99)	ABVIB (n=11)	ADNI (n=133)	AIBL (n=55)	ARWIBO (n=212)	DOD-ADNI (n=247)	EDSD (n=157)	EMIF (n=28)	I-ADNI (n=16)	JADNI (n=122)	PharmaCog (n=153)	PREVENT-AD (n=40)	VITA (n=150)	AD-Mapper (n=325)
**A4**	Fuzzy	-	43%	18%	64%	13%	47%	15%	55%	60%	85%	14%	60%	14%	50%
**ABVIB**	Fuzzy	29%	-	57%	0%	0%	0%	0%	33%	50%	12%	0%	17%	0%	0%
**ADNI**	Fuzzy	15%	71%	-	20%	7%	95%	8%	60%	33%	56%	8%	50%	6%	25%
**AIBL**	Fuzzy	71%	17%	20%	-	39%	37%	33%	44%	50%	39%	44%	56%	47%	14%
**ARWIBO**	Fuzzy	21%	60%	19%	32%	-	7%	97%	44%	36%	23%	100%	21%	100%	23%
**DOD-ADNI**	Fuzzy	41%	40%	94%	49%	11%	-	8%	44%	54%	86%	7%	41%	6%	27%
**EDSD**	Fuzzy	12%	57%	8%	26%	96%	3%	-	40%	71%	16%	100%	29%	99%	30%
**EMIF**	Fuzzy	46%	67%	58%	50%	44%	11%	36%	-	100%	38%	40%	36%	36%	5%
**I-ADNI**	Fuzzy	50%	0%	33%	50%	43%	50%	71%	100%	-	43%	67%	25%	67%	40%
**JADNI**	Fuzzy	85%	62%	50%	45%	35%	88%	44%	71%	57%	-	50%	31%	69%	32%
**PharmaCog**	Fuzzy	12%	57%	7%	29%	99%	3%	100%	50%	100%	24%	-	21%	99%	36%
**PREVENT-AD**	Fuzzy	30%	17%	42%	50%	27%	24%	23%	33%	50%	25%	38%	-	38%	7%
**VITA**	Fuzzy	12%	57%	17%	35%	100%	3%	100%	40%	100%	21%	100%	21%	-	40%

Table S6: Calculated accuracy of the closest cosine similarity of MPNet embeddings for all applicable cohorts in the AD-Mapper variable mappings. The n describes the number of variables that have a corresponding mapping in the AD-Mapper variable mappings.

Mapping from / mapping to	Method	A4 (n=99)	ABVIB (n=11)	ADNI (n=133)	AIBL (n=55)	ARWIBO (n=212)	DOD-ADNI (n=247)	EDSD (n=157)	EMIF (n=28)	I-ADNI (n=16)	JADNI (n=122)	PharmaCog (n=153)	PREVENT-AD (n=40)	VITA (n=150)	AD-Mapper (n=325)
**A4**	MPNet	-	86%	45%	80%	43%	63%	39%	64%	70%	90%	37%	90%	40%	66%
**ABVIB**	MPNet	86%	-	71%	62%	36%	80%	43%	100%	100%	88%	43%	83%	43%	73%
**ADNI**	MPNet	31%	86%	-	53%	23%	96%	24%	100%	67%	62%	23%	50%	24%	60%
**AIBL**	MPNet	86%	83%	53%	-	61%	61%	60%	75%	67%	54%	62%	78%	67%	59%
**ARWIBO**	MPNet	62%	60%	38%	68%	-	36%	96%	61%	43%	41%	99%	79%	100%	61%
**DOD-ADNI**	MPNet	58%	80%	96%	74%	37%	-	28%	61%	77%	87%	25%	77%	27%	56%
**EDSD**	MPNet	61%	57%	37%	70%	98%	38%	-	53%	86%	74%	100%	79%	100%	73%
**EMIF**	MPNet	69%	100%	67%	67%	75%	37%	71%	-	100%	59%	80%	73%	86%	67%
**I-ADNI**	MPNet	50%	100%	0%	67%	57%	50%	71%	75%	-	64%	100%	75%	100%	47%
**JADNI**	MPNet	90%	100%	56%	76%	54%	89%	72%	79%	93%	-	56%	69%	62%	57%
**PharmaCog**	MPNet	58%	57%	37%	62%	100%	36%	100%	62%	100%	65%	-	86%	100%	82%
**PREVENT-AD**	MPNet	80%	83%	50%	72%	73%	57%	69%	78%	75%	62%	77%	-	77%	52%
**VITA**	MPNet	57%	57%	38%	65%	100%	37%	99%	60%	100%	71%	99%	86%	-	80%

Table S7: Calculated accuracy of the closest cosine similarity of OpenAI embeddings for all applicable cohorts in the AD-Mapper variable mappings. The n describes the number of variables that have a corresponding mapping in the AD-Mapper variable mappings.

Mapping from / mapping to	Method	A4 (n=99)	ABVIB (n=11)	ADNI (n=133)	AIBL (n=55)	ARWIBO (n=212)	DOD-ADNI (n=247)	EDSD (n=157)	EMIF (n=28)	I-ADNI (n=16)	JADNI (n=122)	PharmaCog (n=153)	PREVENT-AD (n=40)	VITA (n=150)	AD-Mapper (n=325)
**A4**	OpenAI embeddings	-	86%	90%	73%	50%	91%	61%	73%	80%	87%	60%	100%	43%	66%
**ABVIB**	OpenAI embeddings	86%	-	71%	62%	64%	80%	57%	100%	100%	75%	57%	83%	57%	64%
**ADNI**	OpenAI embeddings	83%	86%	-	47%	68%	95%	85%	90%	100%	81%	83%	50%	69%	65%
**AIBL**	OpenAI embeddings	93%	83%	67%	-	61%	58%	65%	75%	50%	57%	75%	89%	80%	64%
**ARWIBO**	OpenAI embeddings	80%	80%	85%	81%	-	76%	99%	78%	43%	59%	100%	79%	100%	61%
**DOD-ADNI**	OpenAI embeddings	92%	90%	98%	69%	71%	-	84%	78%	77%	91%	83%	86%	70%	69%
**EDSD**	OpenAI embeddings	80%	71%	84%	78%	98%	81%	-	93%	100%	74%	100%	93%	100%	80%
**EMIF**	OpenAI embeddings	77%	100%	92%	67%	81%	58%	86%	-	100%	65%	87%	91%	86%	71%
**I-ADNI**	OpenAI embeddings	60%	100%	67%	67%	79%	71%	100%	100%	-	71%	100%	75%	100%	67%
**JADNI**	OpenAI embeddings	97%	88%	75%	70%	80%	92%	94%	79%	86%	-	81%	69%	85%	67%
**PharmaCog**	OpenAI embeddings	77%	71%	84%	71%	100%	80%	100%	100%	100%	71%	-	93%	100%	82%
**PREVENT-AD**	OpenAI embeddings	70%	67%	67%	72%	77%	48%	77%	78%	75%	56%	85%	-	85%	44%
**VITA**	OpenAI embeddings	79%	71%	85%	75%	100%	84%	100%	80%	100%	79%	100%	93%	-	80%

Table S8: Comparison of the accuracies achieved using all three approaches. 0 indicates equal accuracy across all methods.

Mapping from / mapping to	A4	ABVIB	ADNI	AIBL	ARWIBO	DOD-ADNI	EDSD	EMIF	I-ADNI	JADNI	PharmaCog	PREVENT-AD	VITA	AD-Mapper
**A4**	-	MPNet, OpenAI embeddings	OpenAI embeddings	MPNet	OpenAI embeddings	OpenAI embeddings	OpenAI embeddings	OpenAI embeddings	OpenAI embeddings	MPNet	OpenAI embeddings	OpenAI embeddings	OpenAI embeddings	MPNet, OpenAI embeddings
**ABVIB**	MPNet, OpenAI embeddings	-	MPnet, OpenAI embeddings	MPNet, OpenAI embeddings	OpenAI embeddings	MPNet, OpenAI embeddings	OpenAI embeddings	MPNet, OpenAI embeddings	MPNet, OpenAI embeddings	MPNet	OpenAI embeddings	MPNet, OpenAI embeddings	OpenAI embeddings	MPNet
**ADNI**	OpenAI embeddings	MPNet, OpenAI embeddings	-	MPNet	OpenAI embeddings	MPNet	OpenAI embeddings	MPNet	OpenAI embeddings	OpenAI embeddings	OpenAI embeddings	0	OpenAI embeddings	OpenAI embeddings
**AIBL**	OpenAI embeddings	MPNet, OpenAI embeddings	OpenAI embeddings	-	MPNet, OpenAI embeddings	MPNet	OpenAI embeddings	MPNet, OpenAI embeddings	MPNet	OpenAI embeddings	OpenAI embeddings	OpenAI embeddings	OpenAI embeddings	OpenAI embeddings
**ARWIBO**	OpenAI embeddings	OpenAI embeddings	OpenAI embeddings	OpenAI embeddings	-	OpenAI embeddings	OpenAI embeddings	OpenAI embeddings	MPNet, OpenAI embeddings	OpenAI embeddings	Fuzzy, OpenAI embeddings	MPNet, OpenAI embeddings	0	MPNet, OpenAI embeddings
**DOD-ADNI**	OpenAI embeddings	OpenAI embeddings	OpenAI embeddings	MPNet	OpenAI embeddings	-	OpenAI embeddings	OpenAI embeddings	MPNet, OpenAI embeddings	OpenAI embeddings	OpenAI embeddings	OpenAI embeddings	OpenAI embeddings	OpenAI embeddings
**EDSD**	OpenAI embeddings	OpenAI embeddings	OpenAI embeddings	OpenAI embeddings	MPNet, OpenAI embeddings	OpenAI embeddings	-	OpenAI embeddings	OpenAI embeddings	MPNet, OpenAI embeddings	0	OpenAI embeddings	MPNet, OpenAI embeddings	OpenAI embeddings
**EMIF**	OpenAI embeddings	MPNet, OpenAI embeddings	OpenAI embeddings	MPNet, OpenAI embeddings	OpenAI embeddings	OpenAI embeddings	OpenAI embeddings	-	0	OpenAI embeddings	OpenAI embeddings	OpenAI embeddings	MPNet, OpenAI embeddings	OpenAI embeddings
**I-ADNI**	OpenAI embeddings	MPNet, OpenAI embeddings	OpenAI embeddings	MPNet, OpenAI embeddings	OpenAI embeddings	OpenAI embeddings	OpenAI embeddings	Fuzzy, OpenAI embeddings	-	OpenAI embeddings	MPNet, OpenAI embeddings	MPNet, OpenAI embeddings	MPNet, OpenAI embeddings	OpenAI embeddings
**JADNI**	OpenAI embeddings	MPNet	OpenAI embeddings	MPNet	OpenAI embeddings	OpenAI embeddings	OpenAI embeddings	MPNet, OpenAI embeddings	MPNet	-	OpenAI embeddings	MPNet, OpenAI embeddings	OpenAI embeddings	OpenAI embeddings
**PharmaCog**	OpenAI embeddings	OpenAI embeddings	OpenAI embeddings	OpenAI embeddings	MPNet, OpenAI embeddings	OpenAI embeddings	0	OpenAI embeddings	0	OpenAI embeddings	-	OpenAI embeddings	MPNet, OpenAI embeddings	MPNet, OpenAI embeddings
**PREVENT-AD**	MPNet	MPNet	OpenAI embeddings	MPNet, OpenAI embeddings	OpenAI embeddings	MPNet	OpenAI embeddings	MPNet, OpenAI embeddings	MPNet, OpenAI embeddings	MPNet	OpenAI embeddings	-	OpenAI embeddings	MPNet
**VITA**	OpenAI embeddings	OpenAI embeddings	OpenAI embeddings	OpenAI embeddings	0	OpenAI embeddings	Fuzzy, OpenAI embeddings	OpenAI embeddings	0	OpenAI embeddings	Fuzzy, OpenAI embeddings	OpenAI embeddings	-	MPNet, OpenAI embeddings

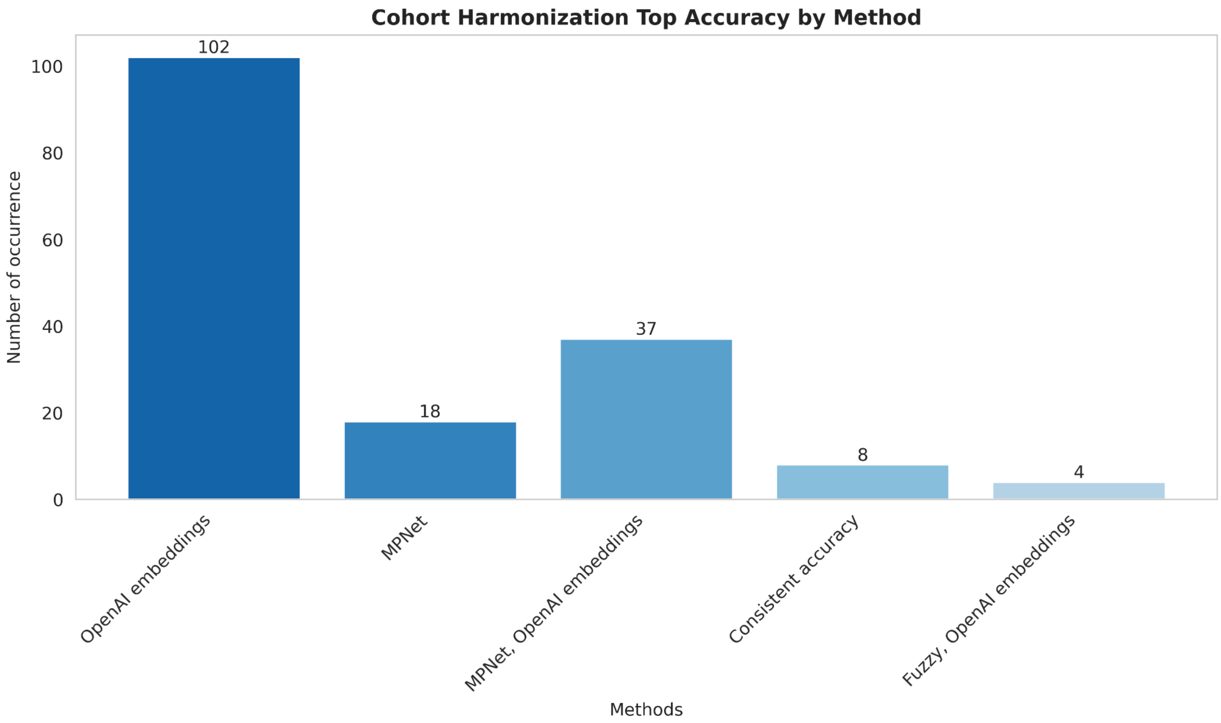

Figure S2: Total occurrences of highest accuracy achieved via different methods for AD cohort harmonization. Note: when two methods are mentioned together, it indicates that both methods achieved the same accuracy, which was superior to the accuracy of the last method mentioned.

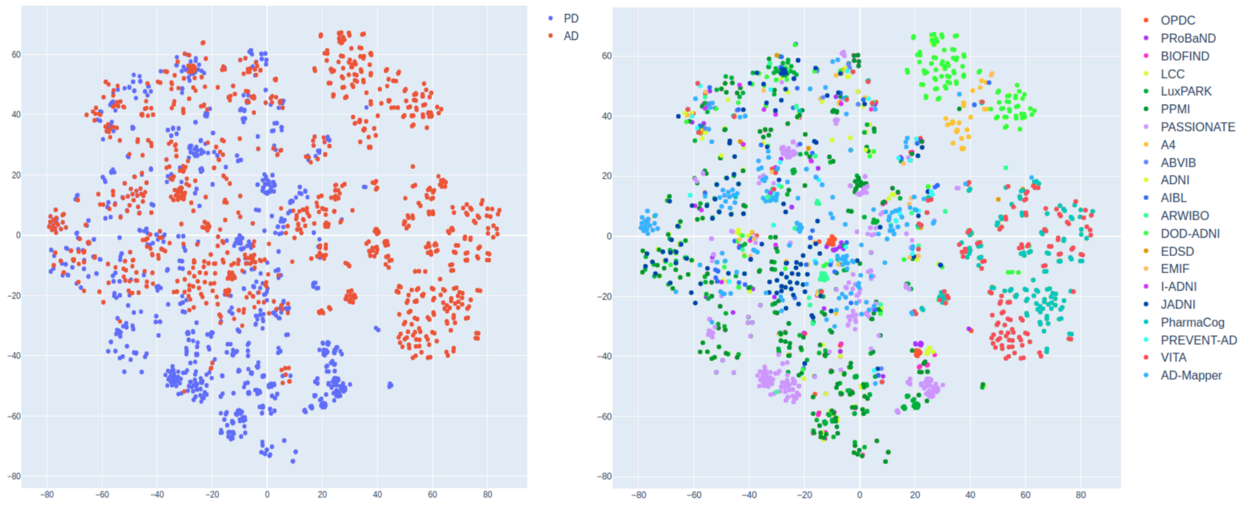

(a) **(b)**

Figure S3: Two-dimensional t-SNE distribution plot of all computed AD and PD variable description embeddings using MPNet. The first plot **(a)** shows the distribution between AD and PD variable descriptions, and the second plot **(b)** the distribution of each cohort.

Accuracy: A to B vs. B to A

By comparing the harmonization results of one cohort to another and rearranging the PD cohorts (e.g., harmonizing PPMI to PRoBaND and PRoBaND to PPMI), we observed that the accuracy remained unchanged or changed marginally in most cases. While harmonizing PRoBaND to OPDC, BIOFIND to OPDC, and PPMI to LuxPARK using OpenAI embeddings, had the same accuracy. The unchanged results could potentially be due to similarities between the cohorts' descriptions. Similarly within AD cohorts, only in 10 cohort-to-cohort harmonization, the accuracy remained the same when the order of cohorts to be harmonized to another was switched.

When harmonizing LCC to LuxPARK, OpenAI embeddings achieved an accuracy of 89%, and harmonizing LuxPARK to LCC resulted in an accuracy of 88%. In a few instances, there was a moderate variation in accuracy, with the maximum change being 15%. Similarly, string matching accuracies differed when the order of cohort that was used for harmonization was switched. However, unlike the OpenAI embedding-based accuracies, the changes within the extracted result varied substantially for half of the cases (i.e., string matching results). One extreme case was observed for the

harmonization of OPDC to PPMI with 7% accuracy whereas the harmonization of PPMI to OPDC had an accuracy of 57%.

REFERENCES

1. Wegner, P. *et al.* Semantic Harmonization of Alzheimer's Disease Datasets Using AD-Mapper. *J. Alzheimers Dis.* **99**, 1409–1423 (2024).
2. Sperling, R. A. *et al.* The A4 study: stopping AD before symptoms begin? *Sci. Transl. Med.* **6**, 228fs13 (2014).
3. Rodriguez, F. S., Zheng, L. & Chui, H. C. Psychometric Characteristics of Cognitive Reserve: How High Education Might Improve Certain Cognitive Abilities in Aging. *Dement. Geriatr. Cogn. Disord.* **47**, 335–344 (2019).
4. Mueller, S. G. *et al.* Ways toward an early diagnosis in Alzheimer's disease: the Alzheimer's Disease Neuroimaging Initiative (ADNI). *Alzheimers Dement. J. Alzheimers Assoc.* **1**, 55–66 (2005).
5. Ellis, K. A. *et al.* The Australian Imaging, Biomarkers and Lifestyle (AIBL) study of aging: methodology and baseline characteristics of 1112 individuals recruited for a longitudinal study of Alzheimer's disease. *Int. Psychogeriatr.* **21**, 672–687 (2009).
6. Frisoni, G. B. *et al.* Markers of Alzheimer's disease in a population attending a memory clinic. *Alzheimers Dement. J. Alzheimers Assoc.* **5**, 307–317 (2009).
7. Weiner, M. W. *et al.* Effects of traumatic brain injury and posttraumatic stress disorder on Alzheimer's disease in veterans, using the Alzheimer's Disease Neuroimaging Initiative. *Alzheimers Dement. J. Alzheimers Assoc.* **10**, S226-235 (2014).
8. Brueggen, K. *et al.* The European DTI Study on Dementia - A multicenter DTI and MRI study on Alzheimer's disease and Mild Cognitive Impairment. *NeuroImage* **144**, 305–308 (2017).
9. Bos, I. *et al.* The EMIF-AD Multimodal Biomarker Discovery study: design, methods and cohort characteristics. *Alzheimers Res. Ther.* **10**, 64 (2018).
10. Cavedo, E. *et al.* The Italian Alzheimer's Disease Neuroimaging Initiative (I-ADNI):

validation of structural MR imaging. *J. Alzheimers Dis. JAD* **40**, 941–952 (2014).

11. Iwatsubo, T. Japanese Alzheimer's Disease Neuroimaging Initiative: present status and future. *Alzheimers Dement. J. Alzheimers Assoc.* **6**, 297–299 (2010).
12. Galluzzi, S. *et al.* Clinical and biomarker profiling of prodromal Alzheimer's disease in workpackage 5 of the Innovative Medicines Initiative PharmaCog project: a 'European ADNI study'. *J. Intern. Med.* **279**, 576–591 (2016).
13. Tremblay-Mercier, J. *et al.* Open science datasets from PREVENT-AD, a longitudinal cohort of pre-symptomatic Alzheimer's disease. *NeuroImage Clin.* **31**, 102733 (2021).
14. Fischer, P. *et al.* Vienna Transdanube Aging 'VITA': study design, recruitment strategies and level of participation. *J. Neural Transm. Suppl.* 105–116 (2002) doi:10.1007/978-3-7091-6139-5_11.
15. Kang, U. J. *et al.* The BioFIND study: Characteristics of a clinically typical Parkinson's disease biomarker cohort. *Mov. Disord.* **31**, 924–932 (2016).
16. Pont-Sunyer, C. *et al.* The prodromal phase of leucine-rich repeat kinase 2-associated Parkinson disease: Clinical and imaging Studies. *Mov. Disord. Off. J. Mov. Disord. Soc.* **32**, 726–738 (2017).
17. Hipp, G. *et al.* The Luxembourg Parkinson's Study: A Comprehensive Approach for Stratification and Early Diagnosis. *Front. Aging Neurosci.* **10**, 326 (2018).
18. Hu, M. Oxford Parkinson's Disease Centre Discovery CohortOPDC Discovery. Dementias Platform UK https://doi.org/10.48532/036000 (2017).
19. Verdi, S., Marquand, A. F., Schott, J. M. & Cole, J. H. Beyond the average patient: how neuroimaging models can address heterogeneity in dementia. *Brain J. Neurol.* **144**, 2946–2953 (2021).
20. Malek, N. *et al.* Tracking Parkinson's: Study Design and Baseline Patient Data. *J. Park. Dis.* **5**, 947–959 (2015).
21. Reich, C. *et al.* OHDSI Standardized Vocabularies—a large-scale centralized reference

ontology for international data harmonization. *J. Am. Med. Inform. Assoc.* **31**, 583–590 (2024).

22. EMBL-EBI. Ontology Lookup Service. https://www.ebi.ac.uk/ols4/index (2023).
